# Innovative principles for sport and event tourism development: a framework for improving the value of the destination while considering social sustainability

**DOI:** 10.3389/fspor.2026.1789278

**Published:** 2026-04-13

**Authors:** Azizjon Anvarovich Qodirov, Dilfuza Bakhriddinovna Urakova, Aziza Bakhodirovna Usmanova

**Affiliations:** Faculty of Economics and Tourism, Bukhara State University, Bukhara, Uzbekistan

**Keywords:** destination competitiveness, event legacy, event leveraging, mega-events, social sustainability, sport tourism, sustainable tourism

## Introduction

Sport and event tourism is a significant and rapidly expanding segment of the global tourism economy (1–3), generating substantial economic activity and employment (4) while simultaneously creating complex social and governance challenges for host communities. The transformative potential of sporting events is illustrated by the 1992 Barcelona Olympic Games, which catalyzed the transformation of an industrial city into a world-class tourism destination attracting millions of visitors annually (5). Similarly, the Boston Marathon generates an estimated $509 million annually for the Massachusetts economy (6). However, these figures, drawn primarily from organizational press releases, should be interpreted as indicative rather than definitive, given well-documented methodological concerns surrounding event economic impact assessments (7, 8).

Economic development potential, however, must be considered alongside serious social sustainability concerns. Displacement, community disruption, and inequitable benefit distribution represent persistent challenges across event hosting contexts (9, 10). Research documents that over two million individuals have been displaced as a result of the Olympic Games since 1988 (11, 12), while Boykoff (13) and others have critically examined the governance failures that allow such outcomes to persist. This tension between economic ambition and social harm poses a fundamental challenge for destination managers and policymakers worldwide.

This opinion article advocates for integrated strategies that position social sustainability and destination value improvement as mutually reinforcing objectives. We organize our argument around three interrelated themes: the need for theoretical integration; lessons drawn from comparative mega-event experiences; and a set of evidence-informed principles for socially sustainable sport event tourism.

## Research approach

This article adopts a conceptual research design grounded in narrative theoretical synthesis, a recognized and independently valuable methodology within tourism and sport management scholarship (14, 15). Conceptual research does not require empirical data collection or statistical analysis; rather, it advances knowledge by integrating, interpreting, and reconceptualizing existing theoretical frameworks and empirical evidence (16, 17). Kirillova and Yang (18) note that conceptual contributions are frequently marginalized within tourism research despite serving an indispensable role in theoretical development and in identifying integrative frameworks that empirical studies alone cannot produce.

The evidence base for this article comprises: (a) foundational theoretical frameworks from destination competitiveness, event leveraging, and event portfolio management literatures; (b) secondary comparative analysis of sustainability outcomes across four mega-event case studies, drawn from published sustainability reports, peer-reviewed scholarship, and institutional documents; and (c) policy documents from the International Olympic Committee and United Nations. No primary data were collected. The article does not claim to report quantitative findings; rather, it advances normative propositions and a conceptual framework intended to guide future empirical research and practical governance design. This approach is consistent with Jaakkola's (19) typology of conceptual contributions, specifically the “theory synthesis” approach, which integrates existing knowledge streams to produce a novel integrative framework.

## Key concepts and definitions

Before proceeding, we clarify three constructs that are central to our argument, as their varied usage in the literature can generate conceptual ambiguity.

Destination value refers to the composite competitive attractiveness of a tourism destination, encompassing its resource endowments, management capabilities, and capacity to deliver satisfying visitor experiences that simultaneously generate economic returns for local stakeholders (20). It is inherently multidimensional, incorporating economic, cultural, and environmental dimensions.

Social sustainability in the context of event tourism denotes the capacity of host communities to maintain or improve their quality of life, social cohesion, and equitable access to resources before, during, and after a major sporting event (11). It encompasses dimensions including displacement prevention, community participation, benefit equity, and cultural preservation.

Innovation adoption refers to the uptake by destination managers and event organizers of novel processes, technologies, governance arrangements, and marketing strategies that enhance the efficiency, inclusivity, and sustainability of event delivery. This encompasses smart venue management, digital community engagement, and institutional innovations such as community benefit agreements (21).

## The need for integrated theoretical approaches

Notwithstanding meaningful progress in destination competitiveness models (20, 22), event leveraging frameworks (19, 23), and sustainability evaluations, the integration of these theoretical traditions remains disjointed in both scholarship and practice. Ritchie and Crouch's destination competitiveness model recognizes unique events as significant attractors, yet does not sufficiently address social equity or community welfare dimensions. Chalip's event leverage model offers strategic insight but was not designed to systematically incorporate sustainability trade-offs. Ziakas's (24) event portfolio theory, building on earlier conceptual foundations by Ziakas and Costa (25, 26), provides comprehensive principles but requires further elaboration in contexts where social sustainability is an explicit governance priority (9).

We contend that an integrated approach — one that treats social sustainability and destination value as complementary rather than competing objectives — is both theoretically grounded and practically necessary. The IOC Olympic Agenda 2020 + 5 (27) and the UN Sustainable Development Goals (28) reflect growing international consensus that destination management organizations must navigate increasingly complex stakeholder expectations. Recent scholarship on strategic communication in sustainable tourism suggests that effective messaging can meaningfully align tourism development with sustainability commitments (29). We propose that these insights, combined with the foundational frameworks above, offer the building blocks for a more coherent integrated approach.

Critically, we acknowledge that existing empirical evidence on the conditions under which economic and social goals converge or conflict remains heterogeneous. Our argument here is normative and theoretical rather than empirical: we advance a position on how destinations ought to approach event governance, grounded in available evidence and theoretical reasoning rather than in original quantitative analysis. Future empirical work — ideally drawing on longitudinal, multi-destination datasets — is necessary to test the conceptual propositions advanced in our framework.

## Lessons from mega-event experiences

Comparative analysis of recent mega-events offers substantial insight into the complex interplay between event hosting and sustainability outcomes. Table 1 summarizes key sustainability initiatives and legacy assessments from four illustrative cases. These cases were selected to represent variation across geographic contexts, governance arrangements, and outcomes, and are drawn from secondary literature, sustainability reports, and prior scholarship rather than primary data collection.

**Table 1:** Comparative analysis of sustainability initiatives and outcomes in recent mega-events.

Mega-Event	Key Sustainability Initiatives	Outcomes and Legacy Assessment
London 2012 Olympics	Brownfield regeneration; sustainable venue design; carbon footprint measurement; zero waste-to-landfill commitment; community sport legacy programs	Strong positive legacy; documented CO2 savings; most sustainability targets achieved; enduring community benefits in East London; widely regarded as a benchmark case (30)
Rio 2016 Olympics	Guanabara Bay cleanup program; Green Passport initiative; urban transport upgrades including metro and BRT systems	Mixed results; environmental pledges substantially unmet; transport infrastructure durable; governance failures and political instability undermined delivery; significant community displacement documented (11)
Tokyo 2020 Olympics	Carbon-neutral commitment; 100% renewable electricity; recycled medals from e-waste; hydrogen-powered Olympic Village	Innovation-focused legacy; circular economy principles demonstrated; COVID-19 disrupted social engagement dimensions; technology transfer benefits realized; no-spectator format limited community participation gains
Qatar 2022 FIFA World Cup	Dismantlable Stadium 974; carbon-neutral event claims; new metro system; hotel sustainability certification requirements	Highly contested; carbon-neutral claims widely disputed by independent analysts; infrastructure improvements noted; severe social concerns regarding migrant worker welfare and human rights overshadowed environmental initiatives (11, 13)

Source: Compiled from Commission for a Sustainable London 2012 (30), Muller et al. (11), Sarvinoz Atoevna et al. (31), Boykoff (13). Cases selected to represent variation in geographic context, governance structure, and sustainability outcomes.

These four cases illustrate that sustainability legacies are shaped not primarily by the ambition of pledges, but by governance capacity, political stability, and the depth of community engagement built into planning processes. London 2012 succeeded in part because sustainability was integrated from the bid stage and subject to independent oversight (30). Rio 2016 and Qatar 2022 demonstrate that ambitious commitments without institutional accountability mechanisms and genuine community inclusion tend to produce uneven or contested results. Research on sustainable hotel operations through mega-event legacies further supports the view that events can serve as catalysts for hospitality sector sustainability improvements — including energy-efficient technologies and staff training — but only when organizational commitment persists beyond the event period (31).

Across all four cases, the most significant governance failures share a common structure: sustainability was treated as an add-on rather than as a foundational design principle. This observation informs our subsequent framework principles.

## Marketing communication and sustainability integration

Effective communication of sustainability programs is essential both for legitimacy and for shaping traveler expectations and behavior. Analysis of mass communication tools in tourism marketing indicates that sustainability standards are increasingly embedded in promotional strategies, reflecting growing consumer awareness (32). UNWTO (33) reports that a substantial majority of global travelers express preference for accommodations and destinations that adopt verifiable sustainable practices, creating both commercial incentives and governance obligations for destination marketers.

Inclusive tourism policies are equally vital to ensuring that the benefits of tourism development are equitably distributed. Research on accommodation inclusivity in emerging tourism economies underscores the necessity of designing tourism infrastructure that actively serves diverse community segments rather than concentrating benefits among existing stakeholders (34). This reinforces our central argument: social sustainability should be treated as a constitutive element of destination development strategy, not as a supplementary concern.

## A framework of principles for socially sustainable sport event tourism

Drawing on the theoretical synthesis and comparative evidence presented above, we advance seven evidence-informed principles for the development of socially sustainable sport event tourism. These principles are normative propositions grounded in existing scholarship; they constitute a conceptual framework intended to guide future empirical investigation and practical governance design rather than claims derived from original quantitative analysis.

1. Portfolio optimization over mega-event dependence. Destinations that manage diverse event portfolios rather than concentrating resources on singular large-scale events are better positioned to sustain long-term competitiveness and community benefits. The Birmingham 2022 Commonwealth Games, delivered within budget while generating substantial societal value (35), illustrates the advantages of integrated portfolio planning over opportunistic mega-event pursuit.
2. Innovation as a competitiveness multiplier. We argue that innovation adoption — spanning process innovations in venue management, marketing innovations in digital community engagement, and institutional innovations in public-private governance — is a key enabling mechanism in transforming event investments into sustained destination competitiveness gains (21). Destinations that treat innovation as peripheral rather than central to event strategy are likely to realize diminishing returns from event hosting.
3. Community engagement as foundational to sustainability. Chalip's (19) social leverage strategy — emphasizing the enhancement of sociability, the creation of event-specific community celebrations, and the cultivation of inclusive participation — offers a theoretically grounded pathway to realizing social sustainability through events. We contend this approach is not merely desirable but necessary for avoiding the social deficits that have characterized poorly governed events.
4. Threshold-sensitive event intensity management. Drawing on the concept of carrying capacity in sustainable tourism (36), we propose that the relationship between event intensity and social sustainability is likely non-linear: moderate levels of event activity may generate community benefits, while excessively dense or intensive event hosting may erode social cohesion and community tolerance. Destination managers should therefore adopt strategic selectivity — prioritizing events with demonstrated community alignment rather than maximizing the volume of hosted events.
5. Regional coordination to generate spatial co-benefits. Event hosting strategies that incorporate regional coordination — sharing infrastructure, visitor flows, and governance learning across neighboring destinations — are more likely to generate broader social and economic benefits than isolated competitive approaches. The trajectory from the Gold Coast 2018 Commonwealth Games to the Brisbane 2032 Olympics illustrates how regional cross-leveraging can create compounding returns (37, 38).
6. Equitable benefit distribution as a social sustainability indicator. We argue, consistent with Owen (39) and Bob and Swart (40), that social sustainability outcomes are more sensitive to how event-generated benefits are distributed than to the aggregate magnitude of those benefits. Governance mechanisms such as local hiring requirements, community benefit agreements, and affordable housing provisions embedded in infrastructure investment represent critical instruments for ensuring community welfare gains.
7. Legacy governance institutionalized from the outset. Pre-event sustainability commitments are necessary but insufficient; their realization depends on formal legacy governance frameworks that include independent monitoring, transparent reporting, and accountability mechanisms sustained beyond the event period. The IOC Olympic Agenda 2020 + 5 (27) and OECD guidelines on event impact measurement provide emerging institutional scaffolding, though implementation varies considerably across host contexts.

These seven principles are designed to be applicable across a range of destination contexts and scales. We recognize that their relative salience will vary depending on institutional capacity, governance arrangements, and the nature of the events pursued. As normative propositions grounded in existing scholarship, these principles are offered as a foundation for governance design and as an agenda for future empirical investigation; they do not themselves constitute or report the findings of quantitative or statistical research.

## Recommendations for destination managers and policymakers

Based on the comparative evidence and conceptual framework presented above, we offer the following targeted recommendations.

Sustainability planning must be incorporated from the initial phases of event bidding rather than treated as a *post-hoc* consideration. Independent oversight mechanisms with transparent, publicly accessible reporting should be established as a precondition of event hosting rights. Realistic and measurable targets should be set based on demonstrated governance capacity rather than aspirational rhetoric.

Post-event legacy management structures must be formally established to ensure that sustainability programs do not dissolve once media attention recedes. For hospitality operators, mega-events represent an opportunity to accelerate existing sustainability transitions — in areas such as energy efficiency, waste reduction, and inclusive employment — generating operational benefits that outlast the event itself.

Policymakers should resist the temptation to evaluate event hosting success primarily through headline economic impact figures, given the well-documented limitations of such assessments (7). A more balanced performance framework — one that integrates indicators of social well-being, community participation, distributional equity, and environmental impact — would provide a more reliable basis for evaluation and accountability.

## Conclusion

Sport and event tourism offers significant prospects for destination development. However, achieving these benefits sustainably requires integrated governance approaches that systematically balance economic, environmental, and social dimensions from the earliest stages of planning. Comparative mega-event evidence demonstrates that sustainability legacies are neither automatic nor uniformly positive: they reflect deliberate choices about governance design, stakeholder inclusion, and accountability mechanisms.

We have advanced a set of seven conceptual principles for socially sustainable sport event tourism, grounded in existing scholarship and comparative evidence. These principles converge on a shared argument: that destinations which treat social sustainability as a core strategic objective — rather than a secondary constraint — are better positioned to realize enduring competitive advantages. Strategic event portfolio management, inclusive community engagement, innovation adoption, and robust legacy governance each contribute to this outcome.

As climate imperatives intensify and social equity concerns become more central to public discourse, the pressure on event organizers and destination managers to demonstrate authentic sustainability will only increase. We encourage future research to pursue rigorous empirical examination of the conditions under which social sustainability and destination competitiveness complement rather than compete with each other — including longitudinal and cross-destination comparative designs that can test the conceptual propositions advanced in this opinion article.
